# Optimization of Silver Nanoparticle Dermal Patch Films for Enhanced Wound Healing: Formulation and Characterization Study

**DOI:** 10.1155/tswj/4800551

**Published:** 2025-07-29

**Authors:** Roisah Nawatila, Astridani Putranti, Celia Susetyo, Elizabeth Masur, Kartini Kartini, Johan Sukweenadhi, Christina Avanti

**Affiliations:** ^1^Department of Pharmaceutics, Faculty of Pharmacy, University of Surabaya, Surabaya, Indonesia; ^2^Department of Pharmaceutical Biology, Faculty of Pharmacy, University of Surabaya, Surabaya, Indonesia; ^3^Center of Excellence for Food Products and Health Supplements for Degenerative Conditions, University of Surabaya, Surabaya, Indonesia; ^4^Department of Plant Biotechnology, Faculty of Biotechnology, University of Surabaya, Surabaya, Indonesia

**Keywords:** dermal patch, green synthesis, *Plantago major* L., silver nanoparticles, wound healing

## Abstract

**Background:** Silver nanoparticles (AgNPs) are known for their potent antibacterial properties, making them suitable for wound healing applications.

**Aims:** This study focuses on formulating AgNPs into dermal patch films (DPFs), leveraging the adhesive properties of the film for the effective delivery of active ingredients.

**Methods:** AgNPs were synthesized through a green synthesis method using *Plantago major* L. Leaf extract as a bioreductant. Five distinct formulations, ranging from AgNP concentration of 0% (control), 0.005%, 0.01%, 0.05%, and 0.10%, were optimized and denoted as Formulas 1–5 (F1–F5), respectively. The films were fabricated by solvent casting method employing a manual film applicator. A variety of evaluations were then performed on the films, including assessments of their physical and chemical characteristics. These characteristics included organoleptic properties, film thickness, folding endurance, surface pH, loss on drying (LOD), crystallinity, the interaction between active ingredients and excipients, the morphological characteristics of the films, and a wound healing study.

**Results:** All formulations resulted in smooth and transparent films. Favorable outcomes were observed in film thickness and surface pH measurements. Formulations F1–F4 demonstrated exceptional folding endurance (> 200 times). This is also affirmed by a reduction in the -OH peak in the Fourier transform infrared (FT-IR) spectrum. Powder X-ray diffraction (PXRD) analysis showed that F1-F4 had adopted an amorphous structure, while F5 retained crystalline AgNPs. The drying process revealed that F5 exhibited the lowest moisture loss. Scanning electron microscope (SEM) imaging displayed distinct morphologies among the five formulations. F4 and F5 exhibited the highest percentage of wound healing.

**Conclusion:** The formulation of AgNPs synthesized through a green synthesis method, utilizing *Plantago major* L. leaf extract as a bioreductant, has demonstrated significant improvements in the physical characteristics, particularly in Formulations F1–F4. Notably, F4 exhibited the highest wound healing efficacy. Therefore, the findings of this study suggest that F4 (AgNPs 0.05%) represents the most promising DPF formulation for enhanced wound healing applications.

## 1. Introduction

Recent advances in nanotechnology and nanoscience have radically changed the way to treat and prevent various diseases. Among several metallic nanoparticles used in biomedical applications, silver nanoparticles (AgNPs) stand out as one of the most important and widely studied [1]. Their notable biological properties include antibacterial activity, anti-inflammatory effects, and wound healing efficacy [2]. Due to their broad-spectrum activity against Gram-positive, Gram-negative, aerobic, and anaerobic microbes, AgNPs can be used as topical antimicrobial agents in the form of silver-containing medications and wound dressings [3]. Generally, the synthesis of AgNPs can be carried out through three main approaches: physical, chemical, and biological methods. Interestingly, biological synthesis offers the advantage of producing AgNPs with high yield, stability, and solubility [4]. Given the various methods of synthesis, the selection of a suitable and sustainable approach becomes crucial in optimizing both the safety and efficacy of AgNPs for biomedical applications.

The green synthesis or biological method was chosen because it is safe, environmentally friendly, simple, and cost-effective [5]. The sources of green synthesis are microorganisms and plants. However, microorganism-mediated synthesis methods present several limitations, including the requirement for professional expertise and aseptic conditions during the production process. Hence, exploring the plant-based method as potential biofactories has gained heightened interest in the biological synthesis of nanoparticles [6]. Plant metabolites, such as proteins, enzymes, tannins, phenols, sugars, and flavonoids, act as reducing or stabilizing agents to form nanometals. Plants that contain large amounts of this compound can be used as good bioreducing agents in metal nanoparticles [7]. Among plant-based sources, certain species have shown promising capabilities in facilitating nanoparticle synthesis through their bioactive compounds.

*Plantago major* L. was selected based on the presence of biologically active compounds that play a critical role in nanoparticle formation, notably by reducing metal salts and functioning as stabilizing agents. Moreover, the use of *Plantago major* L. was supported by its extensive applications in the biomedical field, including anti-inflammatory, antibacterial, antifungal, antioxidant, antitumor, immunomodulatory, antiulcerogenic, and wound healing properties [8]. According to a study conducted by Mahmood et al., the aqueous extract of *Plantago major* L. exhibited wound healing activity in animal models. Therefore, its application as a nanoparticle synthesis medium is expected to provide synergistic effects with the resulting AgNPs, particularly in enhancing antibacterial activity and promoting wound healing. In addition, the water extract of *Plantago major* L. with a concentration of 0.25% at 70°C gave the best combination result because it had an organoleptic form of silver luster and produced the highest yield of 9.13 mg. It also has a particle size of 129.20 nm and a polydispersity index (PDI) value of 0.25 [9]. To harness these properties in a practical and therapeutic form, the synthesized AgNPs from *Plantago major* L. were further developed into a suitable dosage form for wound treatment.

The dermal patches used for wound healing include active ingredients and formulas attached to the skin. In its application, dermal patch technology has proven to be the safest, fastest, and easiest to use. Other advantages include being very comfortable because it is only affixed to the skin and ensures that all wounds are tightly closed and protected from external conditions that worsen the injury. In addition, it can also deliver the medication to a specific target; if unwanted effects occur, they can be stopped immediately by removing the patch [10–12]. To effectively deliver AgNPs via dermal patches, an appropriate manufacturing technique is essential to ensure uniformity, stability, and performance of the patch.

The solvent casting method is often chosen to make dermal patch films (DPFs) because this method is widely preferred, relatively easy, and does not require expensive equipment. This method is carried out by dissolving the active ingredients and excipients in water. The solution is then poured and dried to form a dermal patch resembling a film layer. Therefore, film-forming agents such as hydroxypropyl methylcellulose (HPMC) and Carbomer 974P are essential, as they critically influence the patch's physical and mechanical properties [13].

HPMC is a water-soluble polymer with excellent film-forming properties, producing transparent, biodegradable, nontoxic, and oil-resistant films. It was selected for its inert nature and favorable texture. HPMC dissolves in cold water and forms a thermally reversible, relatively hard gel when heated at 50°C–80°C [14]. Meanwhile, Carbomer 974P is a high molecular weight polymer cross-linked with allyl pentaerythritol, known for its strong bioadhesive and viscoelastic properties. According to the FDA, it is widely used in topical formulation due to its safety and nonirritating nature when applied to the skin [15]. Building on the selected materials and methods, the present study formulated and evaluated DPFs containing various concentrations of AgNPs to assess their wound healing potential.

The aim of this study is to develop the most effective DPF formulation of AgNPs for wound healing, as assessed through the evaluation of its physical and chemical characteristics and its efficacy in promoting wound healing in rat models. In this study, five DPF formulas were developed using various concentrations of AgNPs: 0% (control/F1), 0.005% (F2), 0.01% (F3), 0.05% (F4), and 0.10% (F5). The DPFs were evaluated based on their physical and chemical characteristics, including organoleptic properties, film thickness, folding endurance, surface pH, and loss on drying (LOD). Crystallinity was assessed using powder x-ray diffraction (XRD) (PXRD), while molecular interaction between active ingredients and excipients was analyzed through Fourier transform infrared (FT-IR) spectroscopy. The morphological properties of the patches were observed using scanning electron microscope (SEM). Furthermore, a wound healing study on rat skin was conducted to evaluate the therapeutic efficacy of each formula. Through this comprehensive evaluation, the study is aimed at identifying the most optimal and effective AgNP DPF formulation for wound healing applications.

## 2. Materials and Methods

### 2.1. Materials

The dried leaves of *Plantago major* L. were obtained from the Center for Research and Development of Medicinal and Traditional Medicinal Plants (Tawangmangu, Central Java, Indonesia). The plant identification and certification (No. 1437/D.T/I/2021) were provided by the Center for Information and Development of Traditional Medicine, Faculty of Pharmacy, University of Surabaya. Pharmaceutical-grade AgNO3, glycerin (plasticizer), and trometamol (acid neutralizer) were obtained from Sigma-Aldrich (St. Louis, United States). Pharmaceutical-grade HPMC (Shanghai Honest Chem, China) and Carbomer 974P (Fagron, Greece) served as film formers. Na EDTA (chelating agent) was provided by Merck (Darmstadt, Germany), and distilled water was supplied by the Faculty of Pharmacy University of Surabaya (Surabaya, Indonesia).

### 2.2. Plant Extract Preparation

The dried leaves were pulverized using a Phillips blender (the Netherlands) and sieved through a 20-mesh screen to obtain a uniform particle size distribution. Subsequently, the resulting powder was extracted via ultrasonic-assisted extraction (Branson 1510, Danbury, United States) using 50% ethanol (10% w/v) for 20 min at a frequency of 37 kHz. After filtration through a Whatman filter, the extract was stored at 4°C to preserve its phytochemical stability prior to its use in the green synthesis of AgNPs [4].

### 2.3. Green Synthesis of AgNPs

*Plantago major* L. leaf extract and AgNO_3_ stock solutions were prepared separately and then mixed to obtain final concentrations of 0.25% (w/v) and 1 mM, respectively, for the synthesis of AgNPs. The heating and stirring process was conducted using a hot plate magnetic stirrer at 70°C for 60 min. After adding AgNO_3_, the pH of the solution was adjusted to 10 by the addition of 0.2 M NaOH. The formation of AgNPs was indicated by a visible color change from yellow to reddish-brown. The purification and collection of AgNPs synthesized from *Plantago major* L. extract were conducted through a two-step centrifugation process. The first step, at 2000 rpm for 10 min, was used to remove residual plant materials and large impurities. The supernatant was then subjected to a second centrifugation step at 11,000 rpm for 15 min using a Sorval Biofuge Stratos Centrifuge (Thermo Scientific Nalgene, Rochester, New York, United States), with eight tubes each containing 35 mL of the supernatant. This method was adapted from our previous study, which demonstrated its effectiveness in nanoparticle recovery during scale-up procedures. The resulting AgNP pellets were washed with deionized water to remove any remaining unwanted components and then dried at room temperature until a constant weight was achieved [4].

### 2.4. DPF Preparation

Five distinct concentrations of AgNPs, including 0% (control), 0.005%, 0.01%, 0.05%, and 0.10%, were optimized in Formulas 1–5 (F1–F5), respectively, as indicated in Table 1. The selected AgNP concentrations (0.005%, 0.01%, 0.05%, and 0.1%) were based on previous studies to assess their effectiveness in wound healing [4, 9]. The dermal patch was developed via the solvent casting method, employing a manual film applicator.

Each formula was prepared as a 50-g film casting solution. The AgNPs were dispersed in distilled water to achieve a concentration of up to 10% in the total solution. Subsequently, the dispersed AgNPs were mixed with HPMC, carbomer 974P, glycerin, Na EDTA, and trometamol in a glass beaker and diluted with distilled water until complete dispersion was achieved through magnetic stirring for 60 min at 50°C and 1000 rpm. Once a clear solution had been formed, this solution was sonicated at room temperature for 15 min. Stirring continued using a magnetic stirrer until the following morning at room temperature and 100 rpm to remove trapped air bubbles and produce a clear solution.

The film casting solution was poured on the glass surface using a manual film applicator to a thickness of 120 *μ*m and repeated in two layers. The glass was then placed in an oven for 15 min at 60°C. Once the drying process was completed, the glass was removed, and the DPFs were formed into squares of 1.8 cm × 1.8 cm. These films were stored in an airtight container at room temperature. Films exhibiting air bubbles, cuts, or imperfections were excluded from the study.

### 2.5. Characterization of DPFs

#### 2.5.1. Visual Appearance

This test is conducted through visual observation of the DPFs, including the shape, color, odor, and texture. The results of this test were compared between the five formulations.

#### 2.5.2. Film Thickness

Given that film thickness can influence the rate at which the polymer is absorbed by the body, film thickness is a highly significant characteristic. A micrometer screw gauge was used to measure the uniform thickness of each film at five distinct points on the film. The mean thickness is calculated in accordance with Equation (1) [14]. The optimal film thickness is reported to range from 50 to 1000 *μ*m [15].

(1) $$\begin{array}{c} \text{Mean }\left(X\right)=\frac{\Sigma \text{Xi}}{\text{ni}}=\frac{\text{sum of all the observations}}{\text{total number of observations}}. \end{array}$$

#### 2.5.3. Folding Endurance

The AgNP DPFs have been rigorously tested to ensure they can be folded with the required flexibility to handle the films on the wound surface in a comfortable, safe, and cautious manner. The folding endurance is determined by manually folding the film repeatedly at the same spot until it breaks, and the folding endurance is measured. The best films have a folding endurance value of 300 or above [16]. A higher rating indicates greater mechanical strength [15]. To obtain an average value, the process was repeated three times for each film.

#### 2.5.4. Surface pH

The surface pH is measured using a Laqua 1100 pH meter (Horiba, United States). The DPF is mixed with 5 mL of distilled water at room temperature until it swells or dissolves. The electrode is then placed on the DPF's surface and given 5 min to acclimate before the pH of the surface is determined. The pH range of 5–7.4 is suitable [17].

#### 2.5.5. LOD

The LOD of AgNP DPFs was measured by Hanson QC-21 moisture content balance (Ohaus, United States). The sample was initially weighed (W0), then dried at 105°C for 15 min, and weighed after drying (W1). The LOD percentage was defined in accordance with Equation (2) [18]. This was done in triplicate for each film to obtain an average value.

(2) $$\begin{array}{c} \text{LOD}=\frac{W1-W0}{W0}\times 100. \end{array}$$

#### 2.5.6. Crystallinity of the Films Using PXRD

PXRD (X'Pert P Analytical, the Netherland) was used to determine the crystallinity of the DPFs of each formula compared to the pure AgNPs. The samples are placed into the sample holder. The analysis was performed on the range of 2*θ* from 5° to 50°, with a scanning rate of 1 s [10]. PXRD analysis was performed at the Materials Engineering Laboratory, Sepuluh November Institute of Technology (Surabaya, Indonesia).

#### 2.5.7. IR Spectrum Study

FT-IR studies were conducted to investigate any potential chemical interactions between AgNPs and other excipients present in dermal patches. The FT-IR spectra of pure AgNPs and the DPFs in each formulation were recorded using an FT-IR spectrometer (Shimadzu, Japan). The dry samples were placed in the holder and scanned from 4000 to 400 cm^−1^ at a 4 cm^−1^ resolution and 0.2 cm/s speed [19].

#### 2.5.8. Morphology Using SEM

The surface morphology of AgNPs and the DPFs in each formula was observed by SEM (Hitachi Model TM3000, Japan) at an accelerating voltage of 15 kV. These observations were made at a magnification of 3000 times [20]. SEM analysis was conducted at the Pharmaceutics Department, Jember University (Jember, Indonesia).

### 2.6. Wound Healing Study

Male Wistar rats weighing between 150 and 250 gram were maintained in accordance with standard lighting, temperature, and humidity conditions. Under anesthesia (ketamine hydrochloride, 100 mg/kg body weight, intraperitoneally), the dorsal skin was shaved and cleansed with betadine. A surgical blade was then used to cut up a full wound, measuring roughly 0.5–1 cm, all the way up to the level of subcutaneous adipose tissue. After the wounding procedure, each rat was provided with food, redistilled water, and was placed in a sterile cage. This study involved five groups: a normal control group F1 (0%) and four experimental groups treated with AgNP dermal patch at different concentrations F2 (0.005%), F3 (0.01%), F4 (0.05%), and F5 (0.10%). Each group consisted of four with two additional reserve rats. Twenty-four hours following wound induction, the experimental groups were treated with a DPF in each of the five formulas (F1–F5). The treatment was administered only once on the first day. This study was approved by the ethics committee of the University of Surabaya under Approval Number 213/KE/XI/2021.

A ruler was employed to measure the residual wound on Days 1 and 3 following incision. From these measurements, the length of the wound was calculated based on the elapsed time throughout the treatment period [21]. Equation (3) was employed to calculate the percentage of wound closure. The difference between the initial and final areas of the wounds was used to calculate the degree of contraction [22].

(3) $$\begin{array}{c} \text{Wound healing}\%=\frac{\left(\text{Area of original wound}-\text{Area of actual wound}\right)}{\text{Area of original wound}}\times 100. \end{array}$$

## 3. Results and Discussion

### 3.1. Visual Appearance

**Figure 1:**
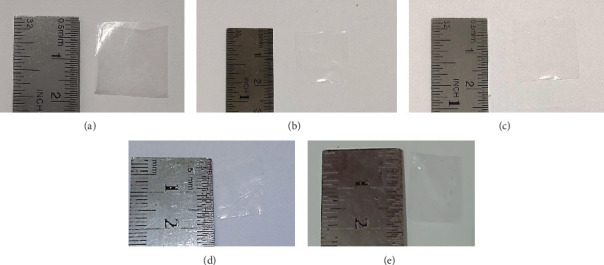
Visual appearance of the AgNPs dermal patch films. (a) F1 with 0% AgNPs, (b) F2 with 0.005% AgNPs, (c) F3 with 0.01% AgNPs, (d) F4 with 0.05% AgNPs, and (e) F5 with 0.10% AgNPs.

The visual observations of the five formulations are presented in Figure 1. Each formulation yielded a flexible, smooth, transparent film that was colorless and odorless, with dimensions of 1.8 cm × 1.8 cm. Figure 1 showed that there were no visual differences among the formulations. This consistency in film appearance, even with varying concentrations of AgNPs, was expected given the nanosized nature of AgNPs and the relatively low concentration range (0.005%–0.10%). The color remained comparable to that of the control film (without AgNPs). At very low concentrations, no visual color change was observed. The overall appearance of the gel remained stable throughout the study.

### 3.2. Film Thickness and Surface pH

The thicknesses of the prepared AgNP DPFs are summarized in Table 2. The thickness ranged from 0.163 ± 0.004 mm for the blank (0% AgNPs) to 0.176 ± 0.004 mm for the DPFs with 0.10% AgNPs. Each formulation was found to be uniform. It was found that the film thickness of the patch increased with increasing AgNP content in film-forming solution. A trend of increasing film thickness with rising AgNP concentration was also observed in the report of Rozilah et al. that an increase in thickness in antibacterial biopolymer composite films occurs as AgNP concentration increases from 0 to 2 wt.% [23].

It is essential to determine the surface pH of the DPFs, as films with highly acidic or basic pH may cause irritation or discomfort upon application or during administration. Table 2 shows that the surface pH of all the formulations (F1–F5) is in the range of 5.79–6.54, indicating a weakly acidic nature. The slight increase in surface pH was attributed to the weakly alkaline nature of the AgNPs, which maintain a pH of approximately 5.4–6.4 during storage. However, all the formulations produced in this study met the surface pH requirements for the DPFs (pH 5.0–7.4) [17]; these films are safe and acceptable to be used on the skin without any problem related to irritation.

### 3.3. Folding Endurance

**Figure 2:**
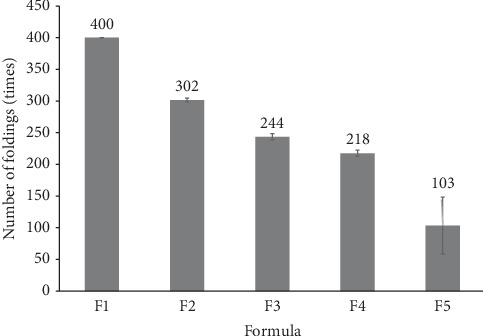
Folding endurance of the AgNP dermal patch films, expressed as the number of times each film can be folded at the same location before breaking.

Figure 2 illustrates the folding endurance of the five formulations. The results demonstrate a negative correlation between AgNP concentrations and folding endurance, with higher AgNPs leading to lower folding endurance. Formulation F1 (control) and F2 (0,005%) AgNPs demonstrated the requisite folding endurance for the DPFs (> 300 folds). However, Formulations F3–F5 did not meet the requirement. Of these, F5 exhibited the lowest folding endurance and produced a brittle film. It is noteworthy that Formulations F1–F4 exhibited a low standard deviation across triplicate measurements, indicating high reproducibility in folding endurance results within these formulations. The brittleness observed in F5, with its low endurance values, is consistent with the findings of other studies in this field. For example, it has been observed that the addition of AgNPs can enhance the mechanical properties of a material up to a specific concentration (e.g., 3.15 wt.%). However, beyond this threshold, further increases in AgNP content (to 4 wt.% or higher) have been observed to result in a reduction in tensile strength and elongation, properties that are closely related to folding endurance. The incorporation of excessive AgNPs may result in an increase in film stiffness, which could potentially lead to a reduction in flexibility and folding endurance [24]. Similarly, research has demonstrated that an increase in the amount of AgNPs from 1 to 4 wt.% has been observed to decrease the ductility of films, which is likely attributed to the formation of nanoparticle agglomerates. This agglomeration may contribute to a reduction in folding endurance, as films become more rigid and less capable of withstanding repeated bending and folding without fracturing [23].

### 3.4. LOD

**Figure 3:**
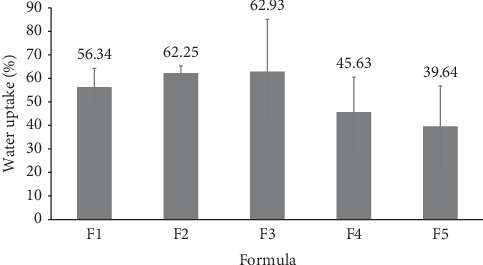
Loss on drying of the AgNP dermal patch films.

The residual water of the AgNP DPFs was evaluated through LOD, which directly impacts the flexibility of the films. The LOD results for Formulations F1–F5 are presented through Figure 3. The findings reveal that the LOD within the dermal patch was notably high, ranging from 39.64% to 62.93%. This indicates that the %LOD exceeds the optimal limit of approximately 12% for patch film [25]. A decreasing trend in LOD values was observed with increasing AgNP concentration in the formula, as higher AgNP levels correlated with reduced water ratios in the formulation. All formulations exhibited high residual water, which contributed to an increased stickiness of the films. This finding indicates that the drying process of the AgNP DPFs may require further optimization to enhance film stability. These findings are consistent with those of a study investigating PVA/chitosan films containing AgNPs and ibuprofen for periodontal treatment. This study observed that AgNPs initially enhanced the films' swelling capacity up to a concentration of 3.15 wt.%. Beyond this concentration, the rate of swelling decreased due to the limited penetration of water, which was attributed to an increase in hydrogen bonding. The restriction in water sorption was accompanied by increased hydrophobicity and a decline in mechanical properties, including tensile strength and elasticity at higher AgNP concentration. These findings highlight the importance of balancing AgNP content to optimize the DPFs with ideal residual water content and mechanical properties.

### 3.5. Crystallinity of the Films Using PXRD

**Figure 4:**
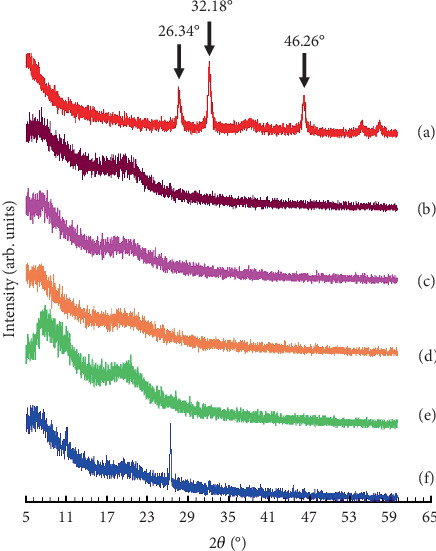
Diffractogram pattern of the AgNPs dermal patch films. (a) AgNPs, (b) F1 with 0% AgNPs, (c) F2 with 0.005% AgNPs, (d) F3 with 0.01% AgNPs, (e) F4 with 0.05% AgNPs, and (f) F5 with 0.10% AgNPs.

The observation of crystallinity was conducted using PXRD, with readings taken at an angle of 2*θ* between 5° and 60°. As illustrated in Figure 4, the crystallinity exhibited a notable enhancement in intensity with the escalation of AgNP concentrations. Furthermore, Formulation F5 demonstrated the persistence of an AgNP peak at an angle of 26.34°, 32.18°, and 46.26°. However, the films of F2–F4 exhibited an amorphous form in comparison to pure AgNPs. Amorphous structures exhibit lower chemical bonding energy between molecular lattices compared to crystalline structures, resulting in enhanced solubility of active ingredients and improved absorption, thereby facilitating a more efficient treatment process [26]. The study utilized XRD to determine the crystalline nature of the AgNPs synthesized using *Plantago major* L. extracts and reported that as the concentration of AgNPs increased, there was a notable enhancement in the intensity of these peaks, suggesting an increase in crystallinity with higher AgNP concentrations. The study also noted that films with lower AgNP concentrations exhibited more amorphous characteristics compared to pure AgNPs, which correlated with enhanced solubility and absorption properties of the films [27].

### 3.6. IR Spectrum Study

**Figure 5:**
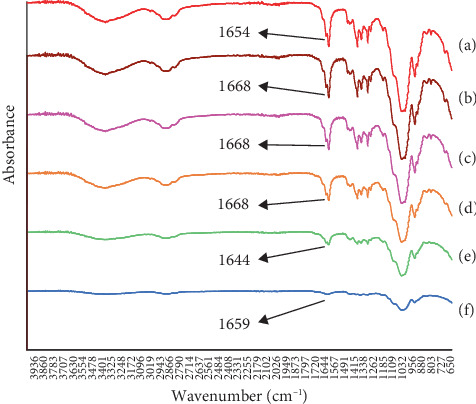
FT-IR spectrum of the AgNPs dermal patch films. (a) AgNPs, (b) F1 with 0% AgNPs, (c) F2 with 0.005% AgNPs, (d) F3 with 0.01% AgNPs, (e) F4 with 0.05% AgNPs, and (f) F5 with 0.10% AgNPs.

The findings of the FT-IR spectrum analysis of AgNPs and DPFs were presented in Figure 5. The FT-IR spectrum of AgNPs as a pure material exhibited a distinctive peak at wavenumber 1654 cm^−1^, which indicates the presence of a carbonyl group [13]. In addition, the spectrum can predict that the AgNP DPFs were successfully formed. It is proven that there was a chemical interaction between AgNPs and the film former (HPMC and Carbomer 974P), so that the DPFs showed the differences in the FT-IR spectrum compared to the pure material. This can be seen from the shift in the characteristic peaks at 1654 cm^−1^ (F1) to 1668 cm^−1^ (F1, F2, and F3), 1644 cm^−1^ (F4), and 1659 cm^−1^ (F5), which predicted that there has been a chemical interaction in the form of hydrogen bonds between AgNPs and the film former (HPMC and Carbomer 974P). The observed changes in the FT-IR spectrum were linked to enhanced solubility and absorption properties of the films, facilitating more effective treatment processes, which support our observations regarding the functional enhancements due to AgNP incorporation [28].

### 3.7. Morphology Using SEM

**Figure 6:**
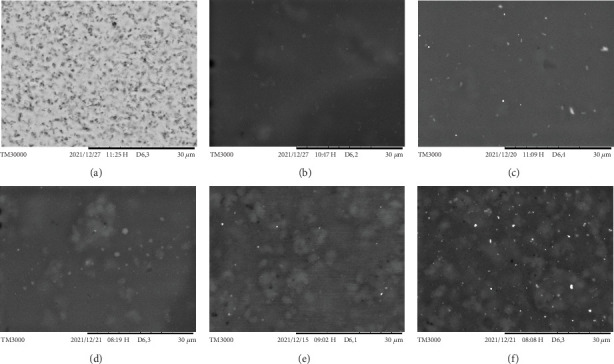
The surface morphology of the AgNP dermal patch films. (a) AgNPs, (b) F1 with 0% AgNPs, (c) F2 with 0.005% AgNPs, (d) F3 with 0.01% AgNPs, (e) F4 with 0.05% AgNPs, and (f) F5 with 0.10% AgNPs.

Figure 6 illustrates the surfaces morphology of AgNPs and DPFs. The results demonstrated that the morphology of AgNPs in a pure state was observed as black particles. Moreover, the surface of F1 (AgNPs 0%) is smoother than that of the other formulations, which may be attributed to the absence of AgNPs and the exclusion of air bubbles that may have been trapped during the manufacturing process due to stirring. In addition, increasing the concentration of AgNPs in F2–F5 will result in a textured surface for the films. This finding is consistent with other evidence indicating that AgNPs exert a pronounced influence on the surface characteristics of thin films. The addition of nanomaterials also affects the properties of HPMC films. The results of the studies indicate that AgNPs appear as black particles and that their incorporation leads to a gritty texture in film. This contrasts with the smoother surfaces observed in formulations without AgNPs, which are thought to result from the trapping of fewer air bubbles during production [29].

### 3.8. Wound Healing Percentage Study

**Figure 7:**
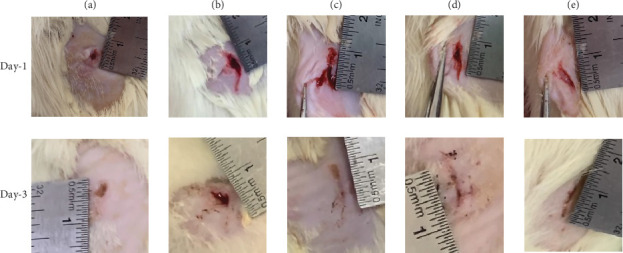
The appearance of the wound in each group on Day 1 to Day 3 during the AgNP dermal patch film effectiveness test. (a) F1 with 0% AgNPs, (b) F2 with 0.005% AgNPs, (c) F3 with 0.01% AgNPs, (d) F4 with 0.05% AgNPs, and (e) F5 with 0.0% AgNPs.

The healing of wounds was evaluated on Day 1 to Day 3, with comparisons made between F1 and F5. On the first day following incision, the AgNP DPFs were applied to the wounds in all groups. By the third day, the scalp had been nearly completely removed, exposing a narrow remaining skin defect (Figure 7). In comparison to other formulations, the experimental group exhibited a more rapid reduction in wound diameter with AgNPs at concentrations of 0.05% (F4) and 0.10% (F5). Moreover, the results of the wound diameter measurements indicated that the wounds in Groups F4 and F5 exhibited a similar size. However, the wound healing of F4 and F5 was significantly higher than that of the other formulas (*p* < 0.05).

The rapid wound closure observed within just 3 days, particularly in the F4 and F5 groups containing 0.05% and 0.10% AgNPs, demonstrates a notable acceleration in the healing process. This is in contrast to other studies, where significant wound reduction typically occurs after 5–7 days of treatment. This rapid healing effect can be attributed to the strong antimicrobial properties of AgNPs, which minimize infection, promote tissue generation, and shorten the inflammatory phase. The efficacy of these AgNP-based dermal patches supports their potential as fast-acting, effective wound healing agents [4].

**Figure 8:**
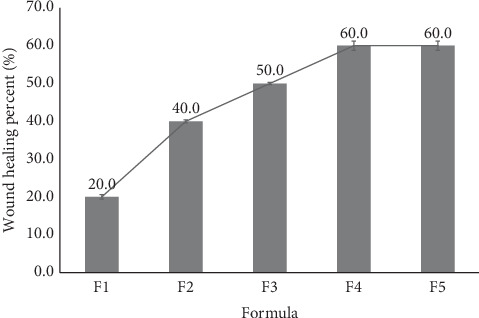
The progression of cutaneous wound healing in rats was evaluated in the following groups: F1 with 0% AgNPs, F2 with 0.005% AgNPs, F3 with 0.01% AgNPs, F4 with 0.05% AgNPs, and F5 with 0.10% AgNPs.

These findings suggest that AgNPs at a concentration of 0.05% (F4) may offer the most effective and optimal wound healing (Figure 8). A study of nanogels that were formulated with AgNPs at various concentrations revealed that the 0.5 mg·g^−1^ (0.05%) AgNP concentration exhibited superior wound healing effects in an incision model, while 0.1 and 1 mg·g^−1^ were effective in excision and burn wound models [30].

## 4. Conclusion

The formulating of AgNPs, synthesized via a green synthesis approach using *Plantago major* L. leaf extract as a bioreductant, into a dermal patch has been demonstrated to effectively enhance the physical characteristics. The films exhibited a smooth and transparent quality across all formulas. The results obtained for film thickness and surface pH were deemed satisfactory. The folding endurance test yielded favorable results regarding reproducibility, as evidenced by F1–F4. The LOD indicated that F5 exhibited the lowest degree of degradation. The PXRD analysis revealed that F1–F4 displayed an amorphous structure, whereas F5 retained a crystalline configuration for the AgNPs. The SEM images demonstrated notable variations in the morphology of the five formulas. F4 and F5 demonstrated the highest percentage of wound healing. The findings of this study suggest that F4 (AgNPs 0.05%) is the most favorable DPF. This formula holds potential for further development into AgNP DPFs with improved physical stability.

## Figures and Tables

**Table 1:** Composition of different film casting solutions.

**Component**	**Function**	**F1 (%)**	**F2 (%)**	**F3 (%)**	**F4 (%)**	**F5 (%)**
AgNPs	Active pharmaceutical ingredients	—	0.005	0.01	0.05	0.10
HPMC	Film former	9.81	9.81	9.81	9.81	9.81
Carbomer 974P	Film former	0.45	0.45	0.45	0.45	0.45
Glycerin	Plasticizer	1.20	1.20	1.20	1.20	1.20
Na EDTA	Chelating agent	0.05	0.05	0.05	0.05	0.05
Trometamol	Acid neutralizer	0.45	0.45	0.45	0.45	0.45
Distilled water^a^	Solvent	ad 100	ad 100	ad 100	ad 100	ad 100

^a^Distilled water was incorporated into the film casting solution until the total weight reached 100% (each formulation was prepared as 50 g).

**Table 2:** Characterization of AgNP dermal patch films.

**Parameter**	**F1 (** **n** = 3**)**	**F2 (** **n** = 3**)**	**F3 (** **n** = 3**)**	**F4 (** **n** = 3**)**	**F5 (** **n** = 3**)**
Film thickness (mm)	0.163 ± 0.004	0.165 ± 0.007	0.172 ± 0.006	0.174 ± 0.002	0.176 ± 0.004
Surface pH	5.79 ± 0.02	6.00 ± 0.08	6.18 ± 0.04	6.34 ± 0.07	6.54 ± 0.08

## Data Availability

The data that support the findings of this study are available from the corresponding author upon reasonable request.

## Supporting Information

Supporting Information Additional supporting information can be found online in the Supporting Information section. The plant identification and certification (No. 1437/D.T/I/2021) were provided by the Center for Information and Development of Traditional Medicine, Faculty of Pharmacy, University of Surabaya.
