# Supplementary material for: Optimization of Silver Nanoparticle Dermal Patch Films for Enhanced Wound Healing: Formulation and Characterization Study
Source: ScientificWorldJournal. 2025 Jul 29;2025:4800551. doi: 10.1155/tswj/4800551 (PMC12324911; doi:10.1155/tswj/4800551)
Supplement: Supporting Information — Additional supporting information can be found online in the Supporting Information section. The plant identification and certification (No. 1437/D.T/I/2021) were provided by the Center for Information and Development of Traditional Medicine, Faculty of Pharmacy, University of Surabaya. [file 4800551.f1.pdf]

UBAYA
UNIVERSITAS SURABAYA
FACULTY OF PHARMACY
PUSAT INFORMASI & PENGEMBANGAN
OBAT TRADISIONAL

No. 1437/D.T/I/2021

Ketua PIPOT Fakultas Farmasi Universitas Surabaya dengan ini menerangkan, bahwa material tanaman yang dibawa oleh Saudara:

Siti Nur Khasanah - 110117421
(Fakultas Farmasi – Universitas Surabaya di Surabaya)

Pada tanggal 11 Januari 2021, ke Pusat Informasi dan Pengembangan Obat Tradisional, berdasarkan buku "Flora Of Java" karangan C.A. Backer Jilid II, halaman 446 mempunyai nama ilmiah sebagai berikut:

Genus	: Plantago
Species	: *Plantago major* (L.)

Klasifikasi tanaman menurut buku *"The Standart Cyclopedia of Horticulture"* karangan L.H. Bailey jilid I (1963) halaman 2-4, adalah sebagai berikut :

Divisi	: Spermatophyta
Sub Divisi	: Angiospermae
Class	: Dicotyledoneae
Sub Class	: Sympetalae
Ordo	: Plantaginales
Family	: Plantaginaceae

Demikian surat keterangan ini dibuat untuk dapat dipergunakan sebagaimana mestinya.

Surabaya, 19 Januari 2021

Lab. Botani Farmasi,

Nikmatul I.E.J, S.Farm., M.Farm-Klin., Apt.
Npk. 215040

Direktur Pusat Informasi & Pengembangan Obat Tradisional,

Kartini, S.Si., M.Si., Apt., Ph.D.
Npk. 203007
